# Supplementary material for: Better individual-level risk models can improve the targeting and life-saving potential of early-mortality interventions
Source: Sci Rep. 2023 Dec 7;13:21706. doi: 10.1038/s41598-023-48888-7 (PMC10709389; doi:10.1038/s41598-023-48888-7)
Supplement: Supplementary file 1 — Supplementary Information. [file 41598_2023_48888_MOESM1_ESM.pdf]

# Better individual-level risk models can improve the targeting and life-saving potential of early-mortality interventions

Chad Hazlett, Antonio Ramos, and Stephen Smith

Table S1: Recall10 results, cross-validation on training set

| Survey | wealth | elastic-logit | rf | xgb | krls | ensemble | efficiency gain |
|---|---|---|---|---|---|---|---|
| Angola 2015 | 0.13 | 0.19 | 0.22 | 0.18 | 0.19 | 0.21 | 1.62 |
| Benin 2011 | 0.15 | 0.31 | 0.27 | 0.29 | 0.31 | 0.32 | 2.16 |
| Burkina Faso 2010 | 0.09 | 0.16 | 0.18 | 0.16 | 0.15 | 0.17 | 1.98 |
| Cameroon 2011 | 0.11 | 0.12 | 0.20 | 0.14 | 0.12 | 0.22 | 2.06 |
| Congo (D.R.) 2013-14 | 0.13 | 0.14 | 0.20 | 0.16 | 0.18 | 0.21 | 1.63 |
| Cote d'Ivoire 2011 | 0.11 | 0.23 | 0.25 | 0.20 | 0.23 | 0.24 | 2.13 |
| Ghana 2014 | 0.15 | 0.20 | 0.12 | 0.17 | 0.17 | 0.18 | 1.22 |
| Guinea 2012 | 0.17 | 0.15 | 0.15 | 0.15 | 0.15 | 0.17 | 1.00 |
| Kenya 2014 | 0.15 | 0.15 | 0.19 | 0.12 | 0.13 | 0.17 | 1.13 |
| Lesotho 2014 | 0.08 | 0.09 | 0.17 | 0.07 | 0.15 | 0.14 | 1.63 |
| Madagascar 2008 | 0.11 | 0.23 | 0.26 | 0.24 | 0.24 | 0.29 | 2.52 |
| Malawi 2016 | 0.10 | 0.16 | 0.20 | 0.16 | 0.18 | 0.19 | 1.91 |
| Mali 2012 | 0.10 | 0.20 | 0.23 | 0.19 | 0.22 | 0.26 | 2.55 |
| Mozambique 2011 | 0.07 | 0.11 | 0.23 | 0.15 | 0.13 | 0.21 | 3.18 |
| Niger 2012 | 0.09 | 0.13 | 0.18 | 0.16 | 0.14 | 0.20 | 2.11 |
| Nigeria 2013 | 0.11 | 0.17 | 0.18 | 0.18 | 0.17 | 0.21 | 1.87 |
| Rwanda 2014 | 0.16 | 0.19 | 0.17 | 0.10 | 0.13 | 0.15 | 0.95 |
| Senegal 2017 | 0.11 | 0.20 | 0.22 | 0.25 | 0.20 | 0.26 | 2.37 |
| Tanzania 2015 | 0.08 | 0.12 | 0.18 | 0.17 | 0.14 | 0.20 | 2.55 |
| Uganda 2016 | 0.14 | 0.16 | 0.17 | 0.17 | 0.17 | 0.16 | 1.19 |
| Zambia 2013 | 0.09 | 0.15 | 0.19 | 0.19 | 0.18 | 0.22 | 2.54 |
| Zimbabwe 2015 | 0.11 | 0.14 | 0.17 | 0.17 | 0.13 | 0.17 | 1.58 |
| Average | 0.12 | 0.17 | 0.20 | 0.17 | 0.17 | 0.21 | 1.90 |

Table S2: Recall20 results, cross-validation on training set

| Survey | wealth | elastic-logit | rf | xgb | krls | ensemble | efficiency gain |
|---|---|---|---|---|---|---|---|
| Angola 2015 | 0.25 | 0.34 | 0.35 | 0.36 | 0.35 | 0.38 | 1.52 |
| Benin 2011 | 0.28 | 0.45 | 0.46 | 0.47 | 0.45 | 0.49 | 1.76 |
| Burkina Faso 2010 | 0.21 | 0.30 | 0.30 | 0.29 | 0.28 | 0.31 | 1.48 |
| Cameroon 2011 | 0.22 | 0.25 | 0.32 | 0.26 | 0.24 | 0.35 | 1.61 |
| Congo (D.R.) 2013-14 | 0.24 | 0.27 | 0.29 | 0.30 | 0.31 | 0.34 | 1.40 |
| Cote d'Ivoire 2011 | 0.20 | 0.37 | 0.33 | 0.37 | 0.36 | 0.42 | 2.04 |
| Ghana 2014 | 0.32 | 0.29 | 0.26 | 0.27 | 0.28 | 0.29 | 0.89 |
| Guinea 2012 | 0.30 | 0.26 | 0.32 | 0.28 | 0.29 | 0.32 | 1.08 |
| Kenya 2014 | 0.28 | 0.26 | 0.30 | 0.21 | 0.26 | 0.30 | 1.07 |
| Lesotho 2014 | 0.17 | 0.26 | 0.28 | 0.16 | 0.26 | 0.24 | 1.42 |
| Madagascar 2008 | 0.26 | 0.37 | 0.37 | 0.39 | 0.37 | 0.43 | 1.68 |
| Malawi 2016 | 0.19 | 0.31 | 0.30 | 0.29 | 0.30 | 0.34 | 1.84 |
| Mali 2012 | 0.20 | 0.34 | 0.36 | 0.36 | 0.36 | 0.38 | 1.88 |
| Mozambique 2011 | 0.17 | 0.22 | 0.35 | 0.30 | 0.28 | 0.34 | 1.93 |
| Niger 2012 | 0.18 | 0.30 | 0.29 | 0.26 | 0.26 | 0.30 | 1.67 |
| Nigeria 2013 | 0.24 | 0.33 | 0.32 | 0.33 | 0.33 | 0.34 | 1.43 |
| Rwanda 2014 | 0.28 | 0.36 | 0.30 | 0.24 | 0.28 | 0.32 | 1.13 |
| Senegal 2017 | 0.28 | 0.35 | 0.33 | 0.34 | 0.36 | 0.36 | 1.27 |
| Tanzania 2015 | 0.15 | 0.22 | 0.29 | 0.27 | 0.24 | 0.29 | 1.87 |
| Uganda 2016 | 0.26 | 0.28 | 0.31 | 0.30 | 0.26 | 0.30 | 1.18 |
| Zambia 2013 | 0.17 | 0.31 | 0.30 | 0.33 | 0.32 | 0.34 | 2.01 |
| Zimbabwe 2015 | 0.20 | 0.27 | 0.33 | 0.31 | 0.25 | 0.33 | 1.66 |
| Average | 0.23 | 0.30 | 0.32 | 0.30 | 0.30 | 0.34 | 1.54 |

Table S3 describes the variables used in the analyses. While the variables shown here include some that we have transformed or processed already, these constitute the raw material from which variables used in the analysis are formed (see description in Materials and Methods and in Table 4 of the main text.)

Table S3: Variable information

| **Variable** | **Type** | **Range of Values** | **Description** |
|---|---|---|---|
| kidbordlog | Numeric | [0.6931,2.9444] | Natural logarithm of the child's birth order |
| pregtermin | Categorical | {NoPregTerm, PregTerm} | Ever had pregnancy terminate |
| agefrstmar | Numeric | [1,49] | Women's age at start of first marriage or union |
| hheadagehh | Numeric | [8,95] | Age of household head |
| urban | Categorical | {Urban, Rural} | Urban or rural living status |
| kidsex | Categorical | {girl, boy} | Sex of child |
| hheadsex | Categorical | {female, male} | Sex of household head |
| edyrtotal | Numeric | [1,25] | Total years educated |
| maternal_age_month | Numeric | [135,584] | Age of mother calculated in months |
| drinkwtr_new | Categorical | {bad, good} | Main source of drinking water |
| floor_new | Categorical | {unsafe, safe} | Main material of floor |
| cookfuel_new | Categorical | {unclean, clean} | Type of fuel used for cooking |
| toilettype_new | Categorical | {flush, pit, unimproved} | Type of toilet |
| religion_new | Categorical | {Buddhist, Christian, Hindu, Other} | Religion of mother |
| wealths | Numeric | [-16.3862,32.4577] | Wealth index categorical score |
| kidbirthmo | Categorical | {1,2,...,12} | Month of the child's birth |
| kidbirthmo_sin | Numeric | [-1,1] | Sine transformation of child's birth month |
| kidbirthmo_cos | Numeric | [-1,1] | Cosine transformation of child's birth month |
| bednetnum | Numeric | {0,1,2,3,4,5,6,7+} | Number of mosquito nets owned by the household |
| bednetnum_log | Numeric | [0.69,2.2] | Natural logarithm of bednetnum+1 |
| prev_death | Categorical | {no, yes} | Mother experienced previous death of child $< 12$mo |
| prev_death_full | Categorical | {no, yes} | Mother experienced previous death of child $< 5$yr |
| district | Categorical | Depends on the country | District where the household lived at the time of survey |
| Province/State | Categorical | Depends on the country | State or Province where the household lived at the time of survey |
| wealthp | Numeric | [0,1] | Wealth percentile based on wealths (by country; unweighted) |
| wealthp2 | Numeric | [0,1] | $\text{wealthp}^2$ |
| malaria_new | Numeric | [0.01,0.74] | Malaria incidence rate from year prior to child's birth |
| mortality.under12m | Categorical | {no, yes} | Outcome variable which records death of the child under 12mo |

# 1 Country-by-Country Results

Efficiency gain is measured by the ratio of recall10 results for each algorithm compared to the recall10 results of the wealth model. Table S4 shows the average results from cross-validation on the training set, and Table S5 shows the results when the trained models are applied to the testing set.

Table S4: Detailed results by country, cross-validation on training set

| Algorithm | recall10 | recall20 | ROC | MRD | MRR | F1 | Efficiency Gain |
|---|---|---|---|---|---|---|---|
| **Angola** | | | | | | | |
| Elastic Net | 0.190 | 0.336 | 0.672 | 0.012 | 1.287 | 0.940 | 1.440 |
| Ensemble | 0.214 | 0.378 | 0.682 | 0.014 | 1.387 | 0.942 | 1.619 |
| KRLS | 0.188 | 0.354 | 0.671 | 0.014 | 1.334 | 0.942 | 1.421 |
| Random Forest | 0.219 | 0.349 | 0.629 | 0.012 | 1.915 | 0.932 | 1.657 |
| Wealth | 0.132 | 0.249 | 0.561 | 0.207 | -5.954 | 0.922 | 1.000 |
| XGB | 0.183 | 0.362 | 0.659 | 0.016 | 1.376 | 0.939 | 1.380 |
| **Benin** | | | | | | | |
| Elastic Net | 0.310 | 0.454 | 0.676 | 0.026 | 1.573 | 0.933 | 2.124 |
| Ensemble | 0.316 | 0.486 | 0.683 | 0.023 | 1.602 | 0.933 | 2.161 |
| KRLS | 0.313 | 0.451 | 0.671 | 0.021 | 1.459 | 0.933 | 2.142 |
| Random Forest | 0.271 | 0.464 | 0.665 | 0.021 | 2.036 | 0.933 | 1.851 |
| Wealth | 0.146 | 0.276 | 0.542 | 0.118 | 2.176 | 0.920 | 1.000 |
| XGB | 0.294 | 0.470 | 0.665 | 0.026 | 1.573 | 0.932 | 2.014 |
| **Burkina Faso** | | | | | | | |
| Elastic Net | 0.161 | 0.301 | 0.609 | 0.008 | 1.119 | 0.921 | 1.826 |
| Ensemble | 0.174 | 0.311 | 0.626 | 0.010 | 1.170 | 0.920 | 1.977 |
| KRLS | 0.154 | 0.284 | 0.611 | 0.009 | 1.133 | 0.920 | 1.751 |
| Random Forest | 0.176 | 0.301 | 0.584 | 0.009 | 1.362 | 0.918 | 1.998 |
| Wealth | 0.088 | 0.209 | 0.552 | 0.147 | 1.944 | 0.918 | 1.000 |
| XGB | 0.164 | 0.289 | 0.611 | 0.013 | 1.186 | 0.920 | 1.865 |
| **Cameroon** | | | | | | | |
| Elastic Net | 0.122 | 0.250 | 0.589 | 0.006 | 1.092 | 0.928 | 1.144 |
| Ensemble | 0.219 | 0.352 | 0.624 | 0.020 | 1.291 | 0.932 | 2.059 |
| KRLS | 0.119 | 0.239 | 0.588 | 0.004 | 1.063 | 0.929 | 1.123 |
| Random Forest | 0.201 | 0.321 | 0.591 | 0.058 | 1.761 | 0.929 | 1.893 |
| Wealth | 0.106 | 0.219 | 0.542 | 0.136 | 1.993 | 0.927 | 1.000 |
| XGB | 0.139 | 0.263 | 0.592 | 0.012 | 1.181 | 0.926 | 1.309 |
| **Congo (D.R.)** | | | | | | | |
| Elastic Net | 0.142 | 0.269 | 0.561 | 0.004 | 1.062 | 0.918 | 1.099 |
| Ensemble | 0.210 | 0.338 | 0.596 | 0.008 | 1.145 | 0.919 | 1.630 |
| KRLS | 0.183 | 0.307 | 0.582 | 0.006 | 1.101 | 0.918 | 1.420 |
| Random Forest | 0.204 | 0.293 | 0.574 | 0.014 | 1.572 | 0.920 | 1.580 |
| Wealth | 0.129 | 0.240 | 0.516 | 0.064 | 34.585 | 0.900 | 1.000 |
| XGB | 0.156 | 0.296 | 0.582 | 0.006 | 1.104 | 0.920 | 1.210 |
| **Cote d'Ivoire** | | | | | | | |
| Elastic Net | 0.232 | 0.374 | 0.633 | 0.017 | 1.208 | 0.908 | 2.046 |
| Ensemble | 0.241 | 0.418 | 0.649 | 0.018 | 1.240 | 0.910 | 2.127 |
| KRLS | 0.226 | 0.362 | 0.636 | 0.018 | 1.223 | 0.909 | 1.993 |
| Random Forest | 0.250 | 0.333 | 0.607 | 0.023 | 1.404 | 0.916 | 2.205 |
| Wealth | 0.113 | 0.205 | 0.530 | 0.076 | 3.141 | 0.911 | 1.000 |
| XGB | 0.202 | 0.374 | 0.604 | 0.014 | 1.175 | 0.906 | 1.784 |

Table S4: Detailed results by country, cross-validation on training set *(continued)*

| Algorithm | recall10 | recall20 | ROC | MRD | MRR | F1 | Efficiency Gain |
|---|---|---|---|---|---|---|---|
| **Ghana** | | | | | | | |
| Elastic Net | 0.203 | 0.295 | 0.543 | 0.002 | 1.043 | 0.928 | 1.348 |
| Ensemble | 0.184 | 0.288 | 0.554 | 0.006 | 1.153 | 0.930 | 1.218 |
| KRLS | 0.170 | 0.281 | 0.559 | 0.000 | 1.007 | 0.928 | 1.127 |
| Random Forest | 0.118 | 0.257 | 0.550 | 0.019 | 1.535 | 0.928 | 0.785 |
| Wealth | 0.151 | 0.323 | 0.554 | -0.038 | 1.012 | 0.894 | 1.000 |
| XGB | 0.170 | 0.269 | 0.559 | 0.005 | 1.111 | 0.929 | 1.127 |
| **Guinea** | | | | | | | |
| Elastic Net | 0.151 | 0.255 | 0.581 | 0.006 | 1.075 | 0.921 | 0.872 |
| Ensemble | 0.174 | 0.325 | 0.623 | 0.010 | 1.146 | 0.920 | 0.999 |
| KRLS | 0.154 | 0.293 | 0.587 | 0.006 | 1.082 | 0.919 | 0.889 |
| Random Forest | 0.155 | 0.322 | 0.605 | 0.016 | 1.374 | 0.917 | 0.891 |
| Wealth | 0.174 | 0.300 | 0.579 | 0.243 | 3.424 | 0.912 | 1.000 |
| XGB | 0.151 | 0.284 | 0.605 | 0.012 | 1.157 | 0.917 | 0.872 |
| **Kenya** | | | | | | | |
| Elastic Net | 0.153 | 0.261 | 0.541 | 0.001 | 1.018 | 0.930 | 1.029 |
| Ensemble | 0.167 | 0.301 | 0.572 | 0.003 | 1.085 | 0.932 | 1.127 |
| KRLS | 0.131 | 0.263 | 0.531 | 0.001 | 1.025 | 0.932 | 0.886 |
| Random Forest | 0.193 | 0.296 | 0.567 | 0.009 | 1.402 | 0.929 | 1.298 |
| Wealth | 0.148 | 0.280 | 0.545 | -0.028 | 0.893 | 0.880 | 1.000 |
| XGB | 0.117 | 0.212 | 0.550 | 0.001 | 1.028 | 0.933 | 0.786 |
| **Lesotho** | | | | | | | |
| Elastic Net | 0.086 | 0.258 | 0.601 | 0.000 | 1.004 | 0.908 | 1.009 |
| Ensemble | 0.138 | 0.240 | 0.582 | 0.005 | 1.089 | 0.919 | 1.625 |
| KRLS | 0.148 | 0.260 | 0.547 | 0.000 | 1.002 | 0.918 | 1.741 |
| Random Forest | 0.173 | 0.283 | 0.592 | 0.017 | 1.420 | 0.921 | 2.036 |
| Wealth | 0.085 | 0.170 | 0.580 | -0.035 | 4.879 | 0.898 | 1.000 |
| XGB | 0.067 | 0.155 | 0.572 | 0.004 | 1.062 | 0.919 | 0.795 |
| **Madagascar** | | | | | | | |
| Elastic Net | 0.228 | 0.375 | 0.633 | 0.014 | 1.279 | 0.935 | 2.001 |
| Ensemble | 0.288 | 0.430 | 0.658 | 0.024 | 1.475 | 0.936 | 2.524 |
| KRLS | 0.236 | 0.370 | 0.644 | 0.013 | 1.267 | 0.935 | 2.072 |
| Random Forest | 0.258 | 0.372 | 0.634 | 0.052 | 1.936 | 0.932 | 2.262 |
| Wealth | 0.114 | 0.256 | 0.529 | 0.126 | 1.530 | 0.918 | 1.000 |
| XGB | 0.245 | 0.394 | 0.655 | 0.018 | 1.361 | 0.937 | 2.143 |
| **Malawi** | | | | | | | |
| Elastic Net | 0.155 | 0.308 | 0.586 | 0.004 | 1.091 | 0.935 | 1.519 |
| Ensemble | 0.195 | 0.344 | 0.619 | 0.008 | 1.209 | 0.935 | 1.909 |
| KRLS | 0.177 | 0.299 | 0.593 | 0.005 | 1.113 | 0.937 | 1.737 |
| Random Forest | 0.204 | 0.299 | 0.591 | 0.015 | 1.779 | 0.933 | 1.997 |
| Wealth | 0.102 | 0.186 | 0.528 | 0.062 | 1.405 | 0.932 | 1.000 |
| XGB | 0.162 | 0.292 | 0.595 | 0.007 | 1.166 | 0.933 | 1.583 |
| **Mali** | | | | | | | |
| Elastic Net | 0.200 | 0.337 | 0.646 | 0.015 | 1.263 | 0.931 | 2.000 |
| Ensemble | 0.255 | 0.382 | 0.667 | 0.026 | 1.429 | 0.933 | 2.553 |
| KRLS | 0.218 | 0.361 | 0.656 | 0.019 | 1.315 | 0.932 | 2.184 |
| Random Forest | 0.226 | 0.358 | 0.633 | 0.053 | 1.798 | 0.929 | 2.263 |
| Wealth | 0.100 | 0.203 | 0.549 | 0.181 | 24.702 | 0.923 | 1.000 |

Table S4: Detailed results by country, cross-validation on training set *(continued)*

| Algorithm | recall10 | recall20 | ROC | MRD | MRR | F1 | Efficiency Gain |
|---|---|---|---|---|---|---|---|
| XGB | 0.195 | 0.361 | 0.655 | 0.017 | 1.297 | 0.929 | 1.947 |
| **Mozambique** | | | | | | | |
| Elastic Net | 0.107 | 0.223 | 0.579 | 0.005 | 1.074 | 0.935 | 1.643 |
| Ensemble | 0.207 | 0.337 | 0.632 | 0.021 | 1.301 | 0.935 | 3.179 |
| KRLS | 0.130 | 0.281 | 0.594 | 0.009 | 1.139 | 0.936 | 2.000 |
| Random Forest | 0.226 | 0.349 | 0.624 | 0.052 | 1.679 | 0.933 | 3.464 |
| Wealth | 0.065 | 0.174 | 0.534 | 0.089 | 2.127 | 0.929 | 1.000 |
| XGB | 0.153 | 0.305 | 0.603 | 0.017 | 1.255 | 0.929 | 2.357 |
| **Niger** | | | | | | | |
| Elastic Net | 0.132 | 0.298 | 0.618 | 0.008 | 1.133 | 0.927 | 1.408 |
| Ensemble | 0.198 | 0.299 | 0.638 | 0.019 | 1.301 | 0.931 | 2.114 |
| KRLS | 0.141 | 0.256 | 0.631 | 0.012 | 1.205 | 0.926 | 1.499 |
| Random Forest | 0.183 | 0.294 | 0.594 | 0.045 | 1.652 | 0.930 | 1.955 |
| Wealth | 0.094 | 0.179 | 0.543 | 0.157 | 3.276 | 0.922 | 1.000 |
| XGB | 0.160 | 0.262 | 0.617 | 0.010 | 1.161 | 0.931 | 1.703 |
| **Nigeria** | | | | | | | |
| Elastic Net | 0.170 | 0.331 | 0.623 | 0.012 | 1.173 | 0.921 | 1.523 |
| Ensemble | 0.209 | 0.343 | 0.638 | 0.018 | 1.245 | 0.923 | 1.867 |
| KRLS | 0.173 | 0.333 | 0.625 | 0.012 | 1.175 | 0.922 | 1.550 |
| Random Forest | 0.176 | 0.322 | 0.609 | 0.035 | 1.429 | 0.920 | 1.576 |
| Wealth | 0.112 | 0.239 | 0.568 | 0.223 | 1.919 | 0.919 | 1.000 |
| XGB | 0.176 | 0.332 | 0.621 | 0.012 | 1.175 | 0.922 | 1.569 |
| **Rwanda** | | | | | | | |
| Elastic Net | 0.186 | 0.358 | 0.626 | 0.004 | 1.123 | 0.937 | 1.194 |
| Ensemble | 0.148 | 0.320 | 0.626 | 0.005 | 1.181 | 0.935 | 0.947 |
| KRLS | 0.134 | 0.282 | 0.618 | 0.004 | 1.129 | 0.935 | 0.856 |
| Random Forest | 0.171 | 0.297 | 0.582 | 0.010 | 1.512 | 0.936 | 1.095 |
| Wealth | 0.156 | 0.284 | 0.569 | 0.194 | 74.088 | 0.934 | 1.000 |
| XGB | 0.103 | 0.236 | 0.569 | 0.003 | 1.091 | 0.934 | 0.658 |
| **Senegal** | | | | | | | |
| Elastic Net | 0.196 | 0.346 | 0.575 | 0.004 | 1.099 | 0.918 | 1.799 |
| Ensemble | 0.258 | 0.361 | 0.597 | 0.034 | 1.830 | 0.928 | 2.367 |
| KRLS | 0.196 | 0.358 | 0.575 | 0.004 | 1.095 | 0.922 | 1.798 |
| Random Forest | 0.221 | 0.333 | 0.575 | 0.064 | 2.294 | 0.929 | 2.025 |
| Wealth | 0.109 | 0.284 | 0.565 | 0.106 | 1.740 | 0.912 | 1.000 |
| XGB | 0.246 | 0.342 | 0.599 | 0.063 | 3.266 | 0.931 | 2.253 |
| **Tanzania** | | | | | | | |
| Elastic Net | 0.121 | 0.223 | 0.569 | 0.003 | 1.065 | 0.936 | 1.544 |
| Ensemble | 0.200 | 0.285 | 0.593 | 0.006 | 1.155 | 0.937 | 2.550 |
| KRLS | 0.136 | 0.242 | 0.573 | 0.003 | 1.079 | 0.934 | 1.741 |
| Random Forest | 0.180 | 0.289 | 0.583 | 0.012 | 1.532 | 0.933 | 2.293 |
| Wealth | 0.078 | 0.153 | 0.548 | -0.127 | 0.371 | 0.932 | 1.000 |
| XGB | 0.168 | 0.270 | 0.579 | 0.005 | 1.114 | 0.935 | 2.145 |
| **Uganda** | | | | | | | |
| Elastic Net | 0.157 | 0.278 | 0.540 | 0.001 | 1.028 | 0.924 | 1.153 |
| Ensemble | 0.163 | 0.304 | 0.572 | 0.002 | 1.064 | 0.929 | 1.192 |
| KRLS | 0.173 | 0.255 | 0.534 | 0.001 | 1.024 | 0.925 | 1.265 |
| Random Forest | 0.172 | 0.307 | 0.564 | 0.001 | 1.649 | 0.929 | 1.264 |

Table S4: Detailed results by country, cross-validation on training set *(continued)*

| Algorithm | recall10 | recall20 | ROC | MRD | MRR | F1 | Efficiency Gain |
|---|---|---|---|---|---|---|---|
| Wealth | 0.136 | 0.257 | 0.525 | -0.021 | 0.840 | 0.913 | 1.000 |
| XGB | 0.170 | 0.299 | 0.577 | 0.005 | 1.112 | 0.931 | 1.247 |
| **Zambia** | | | | | | | |
| Elastic Net | 0.150 | 0.307 | 0.580 | 0.005 | 1.099 | 0.929 | 1.752 |
| Ensemble | 0.217 | 0.344 | 0.617 | 0.009 | 1.196 | 0.933 | 2.545 |
| KRLS | 0.175 | 0.318 | 0.591 | 0.006 | 1.124 | 0.932 | 2.055 |
| Random Forest | 0.186 | 0.300 | 0.587 | 0.016 | 1.590 | 0.931 | 2.180 |
| Wealth | 0.085 | 0.171 | 0.511 | -0.008 | 0.972 | 0.927 | 1.000 |
| XGB | 0.186 | 0.325 | 0.590 | 0.008 | 1.155 | 0.931 | 2.179 |
| **Zimbabwe** | | | | | | | |
| Elastic Net | 0.143 | 0.269 | 0.562 | 0.004 | 1.083 | 0.933 | 1.288 |
| Ensemble | 0.175 | 0.333 | 0.627 | 0.009 | 1.200 | 0.935 | 1.578 |
| KRLS | 0.127 | 0.249 | 0.585 | 0.005 | 1.096 | 0.936 | 1.145 |
| Random Forest | 0.175 | 0.328 | 0.602 | 0.016 | 1.582 | 0.932 | 1.575 |
| Wealth | 0.111 | 0.201 | 0.563 | 2.005 | 4.039 | 0.932 | 1.000 |
| XGB | 0.175 | 0.312 | 0.596 | 0.011 | 1.218 | 0.932 | 1.575 |

Table S5: Detailed results by country, test set

| Algorithm | Recall 10 | Recall 20 | ROC | MRD | MRR | F1 | Efficiency Gain |
|---|---|---|---|---|---|---|---|
| **Angola** | | | | | | | |
| Elastic Net | 0.223 | 0.415 | 0.678 | 0.013 | 1.309 | 0.937 | 1.909 |
| Ensemble | 0.223 | 0.436 | 0.694 | 0.014 | 1.405 | 0.941 | 1.909 |
| KRLS | 0.191 | 0.383 | 0.684 | 0.018 | 1.422 | 0.939 | 1.636 |
| Random Forest | 0.223 | 0.340 | 0.632 | 0.011 | 1.846 | 0.935 | 1.909 |
| Wealth | 0.117 | 0.309 | 0.571 | 0.237 | 6.810 | 0.921 | 1.000 |
| XGB | 0.191 | 0.362 | 0.653 | 0.014 | 1.344 | 0.943 | 1.636 |
| **Benin** | | | | | | | |
| Elastic Net | 0.234 | 0.362 | 0.647 | 0.015 | 1.331 | 0.930 | 1.467 |
| Ensemble | 0.234 | 0.457 | 0.693 | 0.020 | 1.514 | 0.930 | 1.467 |
| KRLS | 0.223 | 0.394 | 0.660 | 0.015 | 1.328 | 0.928 | 1.400 |
| Random Forest | 0.309 | 0.468 | 0.694 | 0.030 | 2.489 | 0.934 | 1.933 |
| Wealth | 0.160 | 0.287 | 0.540 | 0.096 | 1.709 | 0.922 | 1.000 |
| XGB | 0.202 | 0.340 | 0.658 | 0.020 | 1.443 | 0.929 | 1.267 |
| **Burkina Faso** | | | | | | | |
| Elastic Net | 0.153 | 0.353 | 0.620 | 0.008 | 1.118 | 0.922 | 1.278 |
| Ensemble | 0.200 | 0.360 | 0.649 | 0.011 | 1.201 | 0.921 | 1.667 |
| KRLS | 0.147 | 0.407 | 0.639 | 0.011 | 1.166 | 0.920 | 1.222 |
| Random Forest | 0.213 | 0.267 | 0.562 | 0.015 | 1.583 | 0.919 | 1.778 |
| Wealth | 0.120 | 0.233 | 0.554 | 0.182 | 2.126 | 0.918 | 1.000 |
| XGB | 0.160 | 0.313 | 0.639 | 0.012 | 1.178 | 0.921 | 1.333 |
| **Cameroon** | | | | | | | |
| Elastic Net | 0.161 | 0.250 | 0.599 | 0.008 | 1.115 | 0.929 | 2.250 |
| Ensemble | 0.223 | 0.295 | 0.621 | 0.019 | 1.270 | 0.934 | 3.125 |
| KRLS | 0.152 | 0.268 | 0.603 | 0.007 | 1.108 | 0.930 | 2.125 |
| Random Forest | 0.232 | 0.321 | 0.609 | 0.052 | 1.651 | 0.930 | 3.250 |
| Wealth | 0.071 | 0.241 | 0.557 | 0.188 | 2.084 | 0.922 | 1.000 |
| XGB | 0.152 | 0.241 | 0.579 | 0.010 | 1.141 | 0.931 | 2.125 |
| **Congo (D.R.)** | | | | | | | |
| Elastic Net | 0.191 | 0.280 | 0.566 | 0.004 | 1.071 | 0.912 | 1.364 |
| Ensemble | 0.197 | 0.350 | 0.605 | 0.008 | 1.147 | 0.919 | 1.409 |
| KRLS | 0.159 | 0.306 | 0.581 | 0.006 | 1.106 | 0.918 | 1.136 |
| Random Forest | 0.191 | 0.325 | 0.592 | 0.013 | 1.549 | 0.921 | 1.364 |
| Wealth | 0.140 | 0.242 | 0.530 | 0.125 | 5.698 | 0.903 | 1.000 |
| XGB | 0.191 | 0.299 | 0.584 | 0.007 | 1.113 | 0.924 | 1.364 |
| **Cote d'Ivoire** | | | | | | | |
| Elastic Net | 0.286 | 0.476 | 0.633 | 0.018 | 1.221 | 0.914 | 2.400 |
| Ensemble | 0.262 | 0.476 | 0.632 | 0.020 | 1.262 | 0.910 | 2.200 |
| KRLS | 0.274 | 0.405 | 0.604 | 0.016 | 1.204 | 0.910 | 2.300 |
| Random Forest | 0.179 | 0.298 | 0.571 | 0.023 | 1.384 | 0.910 | 1.500 |
| Wealth | 0.119 | 0.202 | 0.536 | 0.107 | -13.687 | 0.915 | 1.000 |
| XGB | 0.286 | 0.476 | 0.655 | 0.022 | 1.273 | 0.912 | 2.400 |
| **Ghana** | | | | | | | |
| Elastic Net | 0.108 | 0.162 | 0.489 | -0.001 | 0.987 | 0.923 | 0.800 |
| Ensemble | 0.189 | 0.243 | 0.556 | 0.009 | 1.212 | 0.926 | 1.400 |
| KRLS | 0.081 | 0.189 | 0.524 | 0.000 | 1.005 | 0.921 | 0.600 |
| Random Forest | 0.216 | 0.216 | 0.534 | 0.032 | 1.904 | 0.929 | 1.600 |
| Wealth | 0.135 | 0.216 | 0.531 | -0.109 | 0.388 | 0.893 | 1.000 |

Table S5: Detailed Results by Country *(continued)*

| Algorithm | Recall 10 | Recall 20 | ROC | MRD | MRR | F1 | Efficiency Gain |
|---|---|---|---|---|---|---|---|
| XGB | 0.162 | 0.297 | 0.550 | 0.004 | 1.094 | 0.931 | 1.200 |
| **Guinea** | | | | | | | |
| Elastic Net | 0.139 | 0.266 | 0.572 | 0.006 | 1.078 | 0.922 | 1.222 |
| Ensemble | 0.190 | 0.329 | 0.627 | 0.012 | 1.181 | 0.921 | 1.667 |
| KRLS | 0.127 | 0.342 | 0.599 | 0.006 | 1.085 | 0.919 | 1.111 |
| Random Forest | 0.215 | 0.380 | 0.628 | 0.025 | 1.621 | 0.914 | 1.889 |
| Wealth | 0.114 | 0.177 | 0.520 | 0.111 | 1.892 | 0.909 | 1.000 |
| XGB | 0.152 | 0.241 | 0.556 | 0.012 | 1.151 | 0.918 | 1.333 |
| **Kenya** | | | | | | | |
| Elastic Net | 0.205 | 0.274 | 0.522 | 0.001 | 1.036 | 0.934 | 1.263 |
| Ensemble | 0.197 | 0.325 | 0.582 | 0.005 | 1.142 | 0.936 | 1.211 |
| KRLS | 0.188 | 0.308 | 0.563 | 0.002 | 1.054 | 0.934 | 1.158 |
| Random Forest | 0.162 | 0.325 | 0.584 | 0.014 | 1.665 | 0.935 | 1.000 |
| Wealth | 0.162 | 0.265 | 0.528 | -0.070 | 0.700 | 0.877 | 1.000 |
| XGB | 0.120 | 0.231 | 0.554 | 0.002 | 1.049 | 0.936 | 0.737 |
| **Lesotho** | | | | | | | |
| Elastic Net | 0.172 | 0.310 | 0.522 | 0.000 | 1.004 | 0.912 | 1.250 |
| Ensemble | 0.172 | 0.241 | 0.566 | 0.003 | 1.048 | 0.918 | 1.250 |
| KRLS | 0.103 | 0.172 | 0.517 | 0.000 | 1.001 | 0.911 | 0.750 |
| Random Forest | 0.138 | 0.276 | 0.564 | 0.005 | 1.156 | 0.906 | 1.000 |
| Wealth | 0.138 | 0.345 | 0.501 | -0.049 | 0.441 | 0.911 | 1.000 |
| XGB | 0.138 | 0.207 | 0.556 | 0.006 | 1.085 | 0.925 | 1.000 |
| **Madagascar** | | | | | | | |
| Elastic Net | 0.099 | 0.264 | 0.553 | 0.004 | 1.080 | 0.925 | 1.125 |
| Ensemble | 0.209 | 0.297 | 0.582 | 0.012 | 1.224 | 0.932 | 2.375 |
| KRLS | 0.132 | 0.264 | 0.567 | 0.005 | 1.106 | 0.928 | 1.500 |
| Random Forest | 0.154 | 0.308 | 0.561 | 0.033 | 1.554 | 0.925 | 1.750 |
| Wealth | 0.088 | 0.187 | 0.491 | 0.032 | 1.140 | 0.912 | 1.000 |
| XGB | 0.099 | 0.242 | 0.571 | 0.004 | 1.091 | 0.928 | 1.125 |
| **Malawi** | | | | | | | |
| Elastic Net | 0.107 | 0.214 | 0.552 | 0.002 | 1.038 | 0.933 | 1.091 |
| Ensemble | 0.170 | 0.277 | 0.599 | 0.006 | 1.158 | 0.938 | 1.727 |
| KRLS | 0.143 | 0.223 | 0.561 | 0.003 | 1.083 | 0.937 | 1.455 |
| Random Forest | 0.196 | 0.304 | 0.629 | 0.011 | 1.639 | 0.935 | 2.000 |
| Wealth | 0.098 | 0.241 | 0.550 | 0.082 | 1.592 | 0.931 | 1.000 |
| XGB | 0.188 | 0.277 | 0.576 | 0.006 | 1.155 | 0.934 | 1.909 |
| **Mali** | | | | | | | |
| Elastic Net | 0.179 | 0.347 | 0.633 | 0.015 | 1.258 | 0.930 | 2.429 |
| Ensemble | 0.232 | 0.389 | 0.653 | 0.023 | 1.379 | 0.933 | 3.143 |
| KRLS | 0.221 | 0.347 | 0.625 | 0.017 | 1.292 | 0.934 | 3.000 |
| Random Forest | 0.211 | 0.337 | 0.626 | 0.044 | 1.647 | 0.928 | 2.857 |
| Wealth | 0.074 | 0.158 | 0.525 | 0.155 | 2.975 | 0.923 | 1.000 |
| XGB | 0.232 | 0.337 | 0.631 | 0.017 | 1.285 | 0.930 | 3.143 |
| **Mozambique** | | | | | | | |
| Elastic Net | 0.075 | 0.168 | 0.571 | 0.003 | 1.052 | 0.934 | 1.000 |
| Ensemble | 0.150 | 0.243 | 0.614 | 0.016 | 1.228 | 0.930 | 2.000 |
| KRLS | 0.103 | 0.252 | 0.586 | 0.009 | 1.134 | 0.929 | 1.375 |
| Random Forest | 0.178 | 0.299 | 0.591 | 0.048 | 1.613 | 0.930 | 2.375 |
| Wealth | 0.075 | 0.187 | 0.504 | -0.060 | 0.501 | 0.931 | 1.000 |

Table S5: Detailed Results by Country *(continued)*

| Algorithm | Recall 10 | Recall 20 | ROC | MRD | MRR | F1 | Efficiency Gain |
|---|---|---|---|---|---|---|---|
| XGB | 0.112 | 0.196 | 0.559 | 0.003 | 1.044 | 0.929 | 1.500 |
| **Niger** | | | | | | | |
| Elastic Net | 0.145 | 0.231 | 0.573 | 0.006 | 1.093 | 0.928 | 1.214 |
| Ensemble | 0.154 | 0.265 | 0.573 | 0.010 | 1.156 | 0.927 | 1.286 |
| KRLS | 0.128 | 0.239 | 0.567 | 0.008 | 1.128 | 0.924 | 1.071 |
| Random Forest | 0.128 | 0.197 | 0.550 | 0.019 | 1.260 | 0.924 | 1.071 |
| Wealth | 0.120 | 0.188 | 0.532 | 0.118 | 2.264 | 0.925 | 1.000 |
| XGB | 0.145 | 0.274 | 0.578 | 0.007 | 1.121 | 0.928 | 1.214 |
| **Nigeria** | | | | | | | |
| Elastic Net | 0.196 | 0.395 | 0.653 | 0.015 | 1.221 | 0.924 | 1.692 |
| Ensemble | 0.249 | 0.401 | 0.667 | 0.022 | 1.305 | 0.927 | 2.154 |
| KRLS | 0.237 | 0.404 | 0.664 | 0.017 | 1.239 | 0.926 | 2.051 |
| Random Forest | 0.211 | 0.353 | 0.619 | 0.041 | 1.507 | 0.924 | 1.821 |
| Wealth | 0.116 | 0.208 | 0.569 | 0.238 | 2.113 | 0.918 | 1.000 |
| XGB | 0.187 | 0.371 | 0.656 | 0.016 | 1.224 | 0.922 | 1.615 |
| **Rwanda** | | | | | | | |
| Elastic Net | 0.212 | 0.303 | 0.618 | 0.004 | 1.140 | 0.937 | 0.875 |
| Ensemble | 0.212 | 0.364 | 0.639 | 0.005 | 1.169 | 0.937 | 0.875 |
| KRLS | 0.212 | 0.364 | 0.597 | 0.004 | 1.124 | 0.938 | 0.875 |
| Random Forest | 0.091 | 0.333 | 0.606 | 0.002 | 1.143 | 0.932 | 0.375 |
| Wealth | 0.242 | 0.485 | 0.651 | 0.399 | 5.757 | 0.937 | 1.000 |
| XGB | 0.212 | 0.364 | 0.637 | 0.008 | 1.256 | 0.939 | 0.875 |
| **Senegal** | | | | | | | |
| Elastic Net | 0.175 | 0.362 | 0.439 | 0.003 | 1.080 | 0.922 | 1.556 |
| Ensemble | 0.325 | 0.412 | 0.628 | 0.035 | 1.849 | 0.934 | 2.889 |
| KRLS | 0.162 | 0.300 | 0.561 | 0.003 | 1.080 | 0.927 | 1.444 |
| Random Forest | 0.275 | 0.375 | 0.619 | 0.075 | 2.545 | 0.933 | 2.444 |
| Wealth | 0.112 | 0.262 | 0.506 | 0.036 | 1.171 | 0.915 | 1.000 |
| XGB | 0.288 | 0.388 | 0.631 | 0.057 | 3.010 | 0.933 | 2.556 |
| **Tanzania** | | | | | | | |
| Elastic Net | 0.190 | 0.317 | 0.573 | 0.003 | 1.066 | 0.940 | 6.000 |
| Ensemble | 0.206 | 0.270 | 0.603 | 0.008 | 1.232 | 0.937 | 6.500 |
| KRLS | 0.159 | 0.254 | 0.549 | 0.002 | 1.059 | 0.935 | 5.000 |
| Random Forest | 0.143 | 0.286 | 0.588 | 0.022 | 1.967 | 0.932 | 4.500 |
| Wealth | 0.032 | 0.111 | 0.542 | -0.133 | 0.318 | 0.934 | 1.000 |
| XGB | 0.143 | 0.333 | 0.592 | 0.007 | 1.167 | 0.935 | 4.500 |
| **Uganda** | | | | | | | |
| Elastic Net | 0.135 | 0.292 | 0.575 | 0.001 | 1.024 | 0.928 | 0.684 |
| Ensemble | 0.250 | 0.417 | 0.658 | 0.004 | 1.124 | 0.937 | 1.263 |
| KRLS | 0.250 | 0.354 | 0.608 | 0.003 | 1.072 | 0.929 | 1.263 |
| Random Forest | 0.240 | 0.333 | 0.596 | 0.003 | 2.662 | 0.937 | 1.211 |
| Wealth | 0.198 | 0.260 | 0.514 | 0.045 | 1.325 | 0.916 | 1.000 |
| XGB | 0.229 | 0.375 | 0.651 | 0.009 | 1.217 | 0.938 | 1.158 |
| **Zambia** | | | | | | | |
| Elastic Net | 0.188 | 0.323 | 0.581 | 0.007 | 1.139 | 0.931 | 1.800 |
| Ensemble | 0.198 | 0.292 | 0.599 | 0.009 | 1.215 | 0.933 | 1.900 |
| KRLS | 0.198 | 0.302 | 0.592 | 0.008 | 1.153 | 0.931 | 1.900 |
| Random Forest | 0.208 | 0.323 | 0.600 | 0.018 | 1.708 | 0.933 | 2.000 |
| Wealth | 0.104 | 0.260 | 0.500 | 0.007 | 1.044 | 0.927 | 1.000 |

Table S5: Detailed Results by Country *(continued)*

| Algorithm | Recall 10 | Recall 20 | ROC | MRD | MRR | F1 | Efficiency Gain |
|---|---|---|---|---|---|---|---|
| XGB | 0.135 | 0.292 | 0.555 | 0.005 | 1.098 | 0.929 | 1.300 |
| **Zimbabwe** | | | | | | | |
| Elastic Net | 0.085 | 0.255 | 0.531 | 0.003 | 1.053 | 0.931 | 1.000 |
| Ensemble | 0.149 | 0.340 | 0.460 | 0.006 | 1.127 | 0.934 | 1.750 |
| KRLS | 0.170 | 0.213 | 0.519 | 0.003 | 1.059 | 0.940 | 2.000 |
| Random Forest | 0.213 | 0.234 | 0.519 | 0.011 | 1.458 | 0.930 | 2.500 |
| Wealth | 0.085 | 0.170 | 0.496 | -0.256 | 0.520 | 0.930 | 1.000 |
| XGB | 0.191 | 0.298 | 0.532 | 0.006 | 1.112 | 0.931 | 2.250 |
